# Recommendations for biomarker data collection in clinical trials by longevity biotechnology companies

**DOI:** 10.1038/s41514-025-00313-1

**Published:** 2025-12-23

**Authors:** Chiara M. S. Herzog, Jesse R. Poganik, Nicola Boekstein, Kristen Fortney, James G. Peyer, Jim Mellon, Risa Starr, Nir Barzilai, Mahdi Moqri

**Affiliations:** 1 Department of Twin Research and Genetic Epidemiology, School of Life Course and Population Sciences, Faculty of Medicine and Life Sciences, King’s College London, London, UK; 2 European Translational Oncology Prevention and Screening Institute, Universität Innsbruck, Innsbruck, Austria; 3 Division of Genetics, Department of Medicine, Brigham and Women’s Hospital, Harvard Medical School, Boston, MA USA; 4 Longevity Biotechnology Association, Ramsey, MN USA; 5 BioAge Labs, Emeryville, CA USA; 6 Cambrian Bio, New York, NY USA; 7 Juvenescence, Ramsey, MN USA; 8 Institute for Aging Research, Albert Einstein College of Medicine, Bronx, NY USA; 9 Department of Genetics, School of Medicine, Stanford University, Stanford, CA USA

**Keywords:** Biomarkers, Medical research

## Abstract

Biomarkers of aging have the potential to transform geroscience clinical trials because of their broad applications in stratifying participants, prioritizing interventions, and monitoring responses to geroprotectors. As longevity biotechnology companies (LBCs) continue to plan and launch innovative clinical trials, standard practices in collecting data and applying biomarkers of aging will allow the field to support parallel and ongoing validation and benchmarking efforts for aging biomarkers. Moreover, defining best practices will ensure future reuse of valuable clinical data through pre-competitive alignment on shared tools. Here, we propose recommendations for such collections. We believe that wide adoption of these recommendations will allow LBCs to produce and leverage the highest quality data from their clinical trials, while also benefiting the geroscience field more broadly with minimal additional effort.

## Introduction

Biomarkers of aging are quantitative parameters that either alone or in composite report on an individual’s biological age^1^. Such biomarkers may be broadly useful in predicting a person’s functional capacity (including physiological, cognitive, or physical), risk of developing aging-associated diseases, or mortality. In the context of gerotherapeutics, biomarkers of aging have enormous potential to accelerate discovery of successful interventions targeting the underlying biology of aging, and to monitor individual effectiveness of such interventions.

Several different types of biomarkers of aging have been proposed. Adopting US Food and Drug Administration (FDA)-Biomarkers, Endpoints, and other Tools (BEST) terminology, these can be classified as molecular, physiological, or digital biomarkers^1^.

Physiological biomarkers of aging, including measures of cardiovascular, metabolic, pulmonary, and physical function, have long been used to assess functional capacity, disease risk, and mortality, and form the foundation of many clinical aging assessments. Computational methods that integrate multiple physiological measures into composite scores (e.g., estimation of biological age^2^) have been widely applied in comparative and population studies of aging and age-related outcomes^3^.

Among molecular biomarkers of aging, omic biomarkers, often referred to as molecular clocks, have attracted considerable interest and currently represent the most actively researched category. Early (‘first generation’) biomarkers of aging were developed by training models to predict chronological age from omic data, such as DNA methylation (DNAm)^4^, metabolomics^5^, or proteomics^6^. Deviations between chronological and predicted age, termed AgeDev^1^, were subsequently found to associate with all-cause mortality and age-related disease^7–9^, and were thus interpreted to represent a readout of biological age. More recently, omic biomarkers have continued to improve in their ability to predict clinical outcomes, reflecting a shift from models trained on chronological age toward models trained directly on aging-associated outcomes such as mortality or health-related endpoints. For example, ‘second generation’ epigenetic clocks (e.g., DNAmPhenoAge, DNAm GrimAge) outperform earlier clocks and represent some of the best predictors of mortality to date^7,8,10^. In parallel, the development of omic biomarkers designed to capture the rate of aging or enriched for features causally associated with aging (also referred to as ‘third generation’) has yielded additional powerful tools^9,11^. Increasingly, the field is also turning toward the integration of multiple data modalities to construct multi-omic biomarkers of aging^12–14^.

Digital biomarkers, enabled by the use of Digital Health Technologies (DHTs) such as wearables, represent a promising yet relatively understudied type of aging biomarker. In contrast to many other biomarkers that are measured at discrete time points due to their dependence on biospecimen collection, digital biomarkers have the ability to conduct continuous monitoring of parameters such as heart rate, sleep, and activity levels. This feature is anticipated to provide more granular functional data, rendering digital biomarkers considerably promising for aging applications^15^.

In the context of longevity biotechnology, continuous development and improvements in biomarkers of aging will be critical to unlock the full potential of geroprotectors. Biomarkers of aging stand to help LBCs in several important ways: to assist in participant stratification; to prioritize candidate interventions; and to provide an early indication that an intervention will increase healthspan and/or lifespan. Although no biomarker of aging has been clinically validated to date, significant advances towards establishing reliable biomarkers of aging have been made in recent years^1,16,17^. One of the key bottlenecks for advancing the validation of biomarkers of aging recently identified by the Biomarkers of Aging Consortium (www.agingconsortium.org) was lack of standardized procedures for collecting specimens/data and applying aging biomarkers^18^. In this paper, we aim to define recommendations for sample and data collection by LBCs running clinical trials, specifically phase I and II trials with small numbers of samples (e.g., *n* < 100). These recommendations include data collection considerations and pre-analytical methods that will ensure data are shareable and comparable among groups. We also cover how biomarker data may best be linked to patient outcomes, and important considerations for patient consent. Broad adoption of these recommended standard collections by LBCs will facilitate the establishment of large, broadly useful datasets for (aging) research that will allow for validation and benchmarking as well as selection of the most promising and useful biomarkers of aging. We anticipate that these recommendations will propel future developments in this field and beyond.

## What to collect in clinical trials to facilitate biomarker research and validation?

In an ideal world, studies would collect as many samples as possible in order to establish a repository of biospecimens and data for research. Several recently completed intervention studies (NCT05678426, ISRCTN83248265), as well as the proposed TAME trial^19^, have collected (or intend to collect) multiple samples, including serum, plasma, blood, urine, stool, and saliva, among others. Although a comprehensive repository is useful, it may not be feasible for collection in all trial protocols. As such, we propose a prioritization framework for assessment of what is most important to collect, based on three main pillars: (1) feasibility, i.e., the effort required for collection in trials; (2) representation, i.e., how representative the sample is of the overall aging process; and (3) range of use, i.e., the number of different analyses that can be conducted with the sample (Table 1). Based on these criteria, we recommend that, at minimum, blood (which provides a snapshot of biological features) and wearable data (which provides continuous information on functional features) should be collected and stored. We note that additional samples may be necessary or useful in specific cases. Samples collected in a standardized manner in early clinical development may support development and validation, but additionally have the potential to be combined with samples and data from other studies, thus significantly advancing biomarker research and geroscience.

**Table 1:** Framework for assessment of sample and data types for standardized collection

Sample	Data type	Timeline	Feasibility	Representation	Range of Use	Examples^a^	Prioritize
Blood	Biological	Snapshot	Routinely collected and often already part of trial protocol	As blood is in constant contact with other tissues and organs, it contains both systemic and organ-specific information which may be leveraged by aging biomarkers	Many omics and specific analyses possible, including DNA (methylation), RNA, proteome, metabolome, lipidome, and cellular analyses; see Table 2 for details	Plasma: EDTA tube (10 ml) DNA: PAXgene Blood DNA Tube (2.5 ml) RNA: PAXgene Blood RNA Tube (2.5 ml)	★
Saliva	Biological	Snapshot	Not typically collected, but easy to collect; limited additional participant burden	Dependent on assay: Possibly less representative than blood for some purposes, but acceptable for others^43,44^	Can be an acceptable source of DNA, but several proteomic platforms require blood rather than saliva	Several commercial products available, such as PAXgene Saliva Collectors (Qiagen) and SafeCollect (Zymo)	
Stool	Biological	Snapshot	Difficult to collect due to inconvenience to participants	Limited primarily to insights into the microbiome and gastrointestinal conditions	Not typically used as standard for most host DNA, proteomic, or metabolomic platforms	Several commercial products available, such as DNA/RNA Shield Fecal Collection Kit (Zymo) and EasySampler Stool Collection Kit (Alpco)	
Urine	Biological	Snapshot	Routinely collected and sometimes already part of trial protocol	Most commonly used for diagnosis of urinary tract infections, diabetes, liver, and kidney disease	Not typically used as standard for most host DNA, proteomic, or metabolomic platforms	Clinical urine collection cups (~100 ml); widely available	
Other biospecimens	Biological	Snapshot	Some types already routinely collected as part of some trials (e.g., buccal or cervical samples)	Provide insights into cell-type-specific features that can be helpful to assess risk for age-related malignancies and risk factors^22^	Not widely used in proteomic or metabolomic assays	For instance, OmniSwab (Qiagen) for buccal swabs; ThinPrep PreservCyt (Hologic) liquid-based cytology for cervical samples; Menstrual cups (Lunette, Diva, Saalt) for menstrual fluid	
Wearable	Functional	Continuous	Not typically collected, but easy to collect; limited additional participant burden	Depending on specific device, can collect several informative functional measures	Possible to collect a range of data, including sleep, heart rate (variability), and activity	Devices are currently available from Apple, Fitbit, Garmin, WHOOP, and many others; choice of device depends on specific parameters desired for study^15^	★

^a^Representative examples that may not be applicable to all studies.

## Blood: collect samples at regular intervals and store appropriately

Blood samples are the most important patient biospecimens for LBCs to prioritize for collection during clinical trials, because: (i) blood samples are typically already collected as part of a clinical trial protocol, reducing the additional burden on trial participants; (ii) blood components originate from several organ systems and may therefore provide insights into the health of both specific organs/systems and overall health, potentially better capturing complex mechanisms associated with aging^20^; (iii) the uses of blood are wide ranging. The broad utility of blood is particularly important given the relatively early stages of biomarker research and the unknowns that still exist regarding the validity of different measures and types of analyses. Various types of blood samples, including whole blood, plasma, serum, or peripheral blood-derived mononuclear cells (PBMCs) outlined in detail in Table 2, represent the gold standard for most current omic measurements and can be used to assess a variety of molecular features, ranging from routine biochemical blood features to current or emerging biomarkers (epigenetic, metabolomic, lipidomic, inflammatory, or other). It is worth mentioning that different omics or biomarker types may exhibit different sensitivity to change over shorter or longer-term periods, which is relevant for the appropriate selection of biomarkers in a given setting, particularly for early clinical trials (phase I, II) that may run over relatively short durations. It has been suggested that proteomic biomarkers or rate of aging epigenetic biomarkers may be more sensitive to short-term interventions.

**Table 2:** Applications of blood samples

Sample type^a^	Example application relevant to biomarkers of aging	Processing required
Whole blood	• Cell counts or functional assays after cell isolation • DNA sequencing • RNA sequencing • DNA methylation profiling	• None; blood is collected in EDTA tubes which may be directly frozen. Peripheral blood-derived mononuclear cells (PBMCs) may be extracted from whole blood.
Serum	• Biochemical blood-based markers for metabolism, liver, kidney, thyroid function, and risk of cardiovascular disease • Inflammatory markers • Proteomics • Metabolomics	• Blood is typically collected in a serum separator tube and allowed to clot for 30–60 min, after which it is separated and serum is isolated.
Plasma	• Proteomics • Metabolomics • Lipidomics • Cell-free DNA or RNA for liquid biopsy	• Blood is typically collected in an anticoagulant treated tube (e.g., EDTA, citrate), after which it is centrifuged and plasma is isolated.

^a^The choice of particular blood sample types is highly study-specific and dependent on the desired downstream assays. For instance, a study aiming to profile DNAm and the plasma proteome might choose to collect both whole blood and plasma to enable these analyses.

To enhance comparability, we recommend that blood collection intervals be standardized based on biomarker type. For more rapidly changing biomarkers, such as proteomic or metabolomic markers, frequent sampling (e.g., weekly or biweekly) may be useful to capture short-term dynamics. Conversely, epigenetic biomarkers, which often reflect cumulative changes and whose trajectories may need longer to change, would benefit from longer intervals between (e.g., monthly or quarterly) to ensure meaningful differences can be detected. The optimal frequency will also depend on the expected effect size of the intervention and the natural variability of the biomarker. Therefore, we propose that clinical trials carefully balance the need for capturing transient changes with the risk of introducing noise due to measurement variability, but further research is required to truly understand longitudinal measurement variability and responsiveness^21^, e.g., including direct longitudinal comparisons of multiple biomarkers in the same cohort. We therefore propose that these samples should be collected at multiple time points, ideally at least monthly - depending on the length of the intervention - and stored appropriately during clinical trials. Importantly, many studies, in particular early-phase clinical trials (phase I and II), already feature standardized collection of blood samples for primary study endpoints such as safety; our recommendation simply extends these by incorporating additional sampling for biomarker research with ideally minimal extra effort. It should be noted that blood samples are not without their drawbacks: several studies have suggested that aging may be cell-type and tissue-specific^20,22,23^, and as a result, blood samples may not be fully representative of aging in other tissues^24^.

To ensure sample and data quality, we suggest the following best practices for blood collection and storage:

1. Blood should be collected from participants via venipuncture before the trial begins and then at regular intervals throughout the duration of the clinical trial. Samples should be collected at the same time of day, preferably after an overnight fast. This is particularly important when measuring metabolites and biochemical markers associated with metabolism that are heavily influenced by recent nutrient intake.
2. Ideally, the maximum volume of blood permissible in the context of the specific trial should be collected to ensure significant volumes for multiple types assessment, and for longer-term storage. We provide some typical volumes based on commonly available collection tubes in Table 1.
3. Samples should be processed as quickly as possible, and maintained on ice or at 4 degrees Celsius prior to storage. Time on ice prior to storage should be as short as possible, to ensure sample integrity and avoid any degradation of biomolecules^25^.
4. Sample types should be processed (e.g., plasma/serum separated out) and aliquoted prior to freezing and storage of samples^26^. Plasma, serum, or other metabolomic samples should be stored at -80 degrees Celsius, and cells should be stored at −150 degrees Celsius (i.e., in liquid nitrogen)^26^. Smaller aliquots are preferable to reduce the number of freeze-thaw cycles samples go through, but this must be balanced with practicality and storage cost considerations.

In addition, special care should be taken during all steps of collection and handling of samples intended for metabolomic and proteomic analyses^27,28^:

1. Samples for metabolomics and proteomics should be immediately processed and stored on dry ice within 2 h of sample collection.
2. Where applicable, e.g., separation of plasma or PBMCs, blood should ideally be processed within 30 min of collection to minimize further metabolism or active/passive transport of analytes between intra-and extracellular compartments, although a period of up to two hours post-collection is acceptable.
3. Metabolite levels are influenced by time of collection, driven by both nutritional status and the circadian rhythm and therefore collection time should be kept constant throughout the trial.

## Wearables: provide devices and collect and store participant biometric data

In addition to collection of blood samples, we recommend prioritizing the collection of biometric data via wearables. Wearables are increasingly capable of monitoring multiple types of biometric parameters, such as activity levels, heart rate variability (HRV), and sleep patterns with a high degree of reliability^9,12–14^. With a decline in movement and activity being one of the most obvious downstream manifestations of aging^2^, it is perhaps unsurprising that emerging evidence suggests the utility of wearable-derived data as biomarkers of aging. Several studies have demonstrated the predictive value of step count, activity, or accelerometer data for disease risk and mortality in cohorts such as NHANES^29,30^ or UK Biobank^31,32^. Moreover, deep neural network models leveraging wearable sensor data have been developed as scalable and cost-effective alternatives to molecular biological age predictors^31–33^. Additionally, HRV^34,35^, sleep duration^36^, and sleep regularity^37^ have been suggested as potential predictors of aging and mortality, though studies using wearable-derived data in this context remain limited.

Popular wearable platforms for clinical trials include Fitbit, Garmin, Apple Health, Polar, and Misfit. These platforms are increasingly deployed in clinical trials listed on ClinicalTrials.gov, and some now provide specific developer options to retrieve, store, and process data^20,23^. Emerging providers such as Oura have focused on specific features, particularly sleep tracking. Given the variety of devices available - recently reviewed by Lu et al.^38^ and Materia and Smyth et al.^39^ - we recommend prioritizing measures of activity levels (e.g., daily steps, active kilocalories), HRV, and sleep, while also considering other potentially valuable parameters depending on the specific clinical trial context.

Several factors must be considered to optimize the use and utility of wearable data:

- *Device type and placement:* The choice of wearable device and its placement (e.g., wrist, finger, or hip) may significantly impact data quality and comparability, with prior studies showing that results from different placements are not directly interchangeable^40,41^. Therefore, it is essential to standardize placement in study protocols and select validated devices appropriate for intended measurements.
- *Key variables collected:* Standardization of measured parameters can improve cross-study comparability; while wearables can capture an enormous diversity of data, we recommend prioritizing measures such as step counts, raw accelerometer data, HRV, sleep duration and phases, and distinctions between sedentary and active periods. Where feasible, access to raw data should be provided to facilitate potential re-analysis and comparison using different algorithms.
- *Duration of wear*: Wearables should be worn for a sufficiently long period to ensure representativeness. A minimum of seven days continuous wear is typically required to obtain stable estimates, though longer durations or repeated assessments may be required. We also recommend collection of baseline data prior to any intervention to gain insights into values at baseline.

We note that practicality of wearable devices is also a major aspect to consider in clinical trials: user experience, comfort, and ease of use influence compliance, and wearables requiring frequent charging may lead to data loss. As such, simpler devices with long battery lives may be favored (e.g., Axivity AX3). Additionally, understanding patient perspectives can inform best practices. To support further standardization, we refer to an in-depth review of digital biomarkers^38^, providing comprehensive guidance on the use of wearables for aging research.

## Track a wide range of age-related health outcomes and conditions and link these to patient samples/data

In addition to the importance of collecting biological and wearable data, it is essential to collect corresponding participant information. Associated data should include detailed demographic information and the medical history of the participant and their family. Irrespective of the indication under assessment in the trial, a broad range of health outcomes corresponding to age-related disease (e.g., cardiovascular diseases, dementia, and cancers) should be monitored, balancing additional trial complexity and data richness, and also considering the length of the study. Other aging-associated outcomes including multimorbidity, performance-based measures of physical and cognitive function, and frailty, measures of disability in activities of daily living (ADLs), and instrumental activities of daily living (IADLs) should be considered when available. Likewise, a detailed record of all concomitant prescribed medication and additional supplementation should be collected. Some existing or planned trials can provide guidance into relevant endpoints to assess. For instance, the planned TAME trial’s primary endpoint is time to incidence of new age-related conditions, defined as myocardial infarction, coronary heart disease, cancer (excluding non-melanoma skin cancer and prostate cancer), mild cognitive impairment/dementia or death, and secondary endpoints are declines in mobility or cognitive function^19^. In the case of long-term follow-up, it is recommended to continue to record information on a wide range of age-related conditions to provide a valuable source of longer-term data.

## Ensure consent for future use of samples and data is established upfront

Informed patient consent is an essential part of all clinical trial protocols, ensuring the subjects understand the purpose of a trial, what data will be collected and processed, and their responsibilities and rights as a study participant, including the ability to cease participation at any time. Beyond the minimum consent requirements for clinical trials, given the enormous value of sample and data collections for biomarker research it is becoming increasingly important to obtain explicit consent for potential future activities without the need to re-engage participants for consent once the trial has been completed.

Given the nascent stage of research into biomarkers of aging, consent should be broad enough to allow for a wide scope of research and analytics, which may not be well defined today, while still being narrow enough for the patient to be considered informed^42^. 23andMe provides a case study of an organization that has obtained broad consent from their participants for storage of samples and use of data in research (https://www.23andme.com/about/consent/). Learnings from 23andMe indicate that when designing your consent document, it is important to not only include consent for both biobanking and future research but also consider governance and ownership of such data and biobanks. Consent for biobanking can be designed to obtain permission for the LBC conducting a clinical trial or contractors to access and analyze stored samples at a later date. The research consent form, including a specification for individual data sharing consent, ensures consent for the use and sharing of de-identified individual-level data (biological, biometric, health outcomes based and self-reported) in research projects to better understand the impact of intervention on the biomarkers of aging. It is important to note that even with appropriate informed consent, broad data sharing may be restricted by law in some countries/regions. Nevertheless, ensuring consent is established upfront is essential to maximize the value of the samples and data collected.

## Conclusion

As biomarkers of aging continue to evolve, standardization and benchmarking are essential to their eventual validation as clinically useful tools to track geroscience interventions. Here we provide recommendations for LBCs and researchers running clinical trials collecting samples and data from trial participants:

1. Collect blood samples at regular intervals from trial participants and store appropriately
2. Provide wearables for participants and collect and store participant biometric data
3. Track a wide range of age-related health outcomes and link these to patient samples and data
4. Ensure consent for future uses of samples and data is established upfront

We envision that by leveraging these recommended standardized collections and by aligning on common tools in a pre-competitive manner, the field can move one step closer toward our goal of a validated, interoperable set of aging biomarkers that inform research and development decision-making by LBCs and ultimately support regulatory approval of biomarkers of aging as surrogate endpoints.

## Data Availability

No datasets were generated or analysed during the current study.
